# Construction of a core collection and SNP fingerprinting database for Chinese chive (*Allium tuberosum*) through Hyper-seq based population genetic analysis

**DOI:** 10.3389/fpls.2025.1603210

**Published:** 2025-07-16

**Authors:** Huamin Zhang, Yanlong Li, Taotao Li, Fangfang Yan, Taotao Fu, Chunli Liao, Dongxiao Liu, Yutao Zhu, Mei Zhao, Peifang Ma, Lianzhe Wang

**Affiliations:** ^1^ College of Life Sciences and Engineering, Henan University of Urban Construction, Pingdingshan, China; ^2^ Center of Healthy Food Engineering and Technology of Henan, Henan University of Urban Construction, Pingdingshan, China; ^3^ Henan Engineering Research Center for Chinese Chive, Pingdingshan Academy of Agricultural Sciences, Pingdingshan, Henan, China

**Keywords:** Chinese chive, genetic diversity, population structure, core collection, DNA fingerprinting

## Abstract

Chinese chive (*Allium tuberosum* Rottler ex Sprengel), an autotetraploid vegetable cultivated in Asia for over 3,000 years, possesses apomictic characteristics. However, issues like intricate genetic admixture and unclear phylogenetic relationships pose challenges for effective germplasm preservation and breeding advancements. In this research, we systematically assessed population structure, constructed a core collection, and developed a DNA fingerprinting system utilizing Hyper-seq sequencing data. Our Hyper-seq-based genotyping revealed 291,547 single nucleotide polymorphisms (SNPs) and 116,223 insertions/deletions (InDels). Population genetic analysis indicated that the 100 A*. tuberosum* accessions can be categorized into two distinct genetic subgroups. These subgroups partially aligned with previously recognized phenotypic classifications based on dormancy traits, underscoring the complex relationship between genetic divergence and adaptive phenotypic variation. A core collection consisting of 22 accessions (22% of the total) was created, maintaining 90.17% of the original genetic diversity. Additionally, we established a DNA fingerprinting system for all 100 accessions using 14 diagnostic SNP markers. This study marks the first comprehensive integration of SNP and InDel markers in systematic analysis of *A. tuberosum* genetic diversity, offering valuable resources for germplasm identification and marker-assisted breeding. These findings deepen the understanding of the genetic architecture of *A. tuberosum* and lay the foundation for molecularly driven breeding strategies.

## Introduction

Chinese chive (*Allium tuberosum* Rottl. ex Spreng), a valuable perennial vegetable, is widely cultivated throughout Asia, particularly in countries such as China, Japan, Korea, and India (Zhou et al., 2015; Pandey et al., 2014). With a cultivation history exceeding three thousand years in China, this plant is celebrated for its distinctive flavor and abundant bioactive components, including sulfur-containing compounds (like *S*-alk(en)ylcysteine sulfoxides), sterols, and polyphenolic flavonoids (Gao et al., 2018). Growing pharmacological research underscores its potential therapeutic applications in cancer chemoprevention, cardiovascular health, immune modulation, glycemic control, antimicrobial effects, and support for the neuro-hepatic system (Powolny and Singh, 2008; Yoshimoto and Saito, 2019). Currently, the germplasm collections of *A. tuberosum* largely consist of landrace varieties and wild genetic resources. However, the unregulated exchange of germplasm and introductions from different regions have created significant issues, such as genetic mixing, unclear lineage documentation, and unresolved phylogenetic relationships. These challenges severely compromise effective germplasm conservation efforts and obstruct breeding advancements. While traditional diversity assessments rely on phenotypic trait analysis (Li et al., 2020a; Zhang et al., 2023), their effectiveness is limited by environmentally influenced trait variability and labor-intensive field evaluations. This critical gap underscores the pressing need for reliable molecular markers to facilitate accurate genetic characterization and of germplasm fingerprinting systems development.

*A. tuberosum* is among the few crops known for facultative apomixis, with more than 90% of its seeds being exact clones of the maternal parent (Zhang et al., 2020). This apomictic reproduction creates a unique dilemma in *A. tuberosum* breeding. While the clonal propagation method poses challenges for germplasm identification and cultivar protection, it also enables the rapid stabilization of desirable traits. The current breeding issues for *A. tuberosum* primarily involve two key areas: (1) the need to swiftly and effectively identify a limited number of sexually propagated seedlings in order to capture novel phenotypic variations, and (2) traceability problems arising from extensive germplasm exchanges that have muddied genetic backgrounds and geographic origins. To tackle these challenges, the development of molecular markers for germplasm identification and selection of sexually reproduced progeny is essential. Although reference genomes for significant *Allium* species like garlic (*A. sativum*), Welsh onion (*A. fistulosum*), and onion (*A. cepa*) have been published (Sun et al., 2020; Liao et al., 2022; Hao et al., 2023), establishing a crucial basis for molecular marker development, *A. tuberosum* remains the only species in this agronomically important genus that lacks genomic characterization. This autotetraploid species (2n=4x=32) possesses an exceptionally large genome (>30 Gb), whose genomic complexity is further complicated by its polyploid structure and high content of repetitive sequences (Zhou et al., 2015; Gohil and Koul, 1983). These factors collectively present substantial obstacles to the molecular breeding of *A. tuberosum*. Currently, expressed sequence tag-simple sequence repeats (EST-SSR) markers derived from transcriptomic studies of *A. tuberosum* (Zhou et al., 2015; Tang et al., 2017; Li et al., 2020b) remain the only molecular resources available for this crop. However, their application in large-scale breeding initiatives is limited due to insufficient marker density and genomic coverage. To overcome this genomic complexity challenge, a systematic application of next-generation sequencing (NGS) technologies is crucial for creating genome-wide molecular markers with multi-allelic resolution, thus facilitating marker-assisted selection (MAS) in *A. tuberosum* breeding programs.

Hyper-seq technology, developed by Zou and Xia in 2022, signifies a groundbreaking advancement in the preparation of sequencing library and multiplex genotyping. It is characterized by its affordability, scalability, and suitability for complex genomes, making it particularly valuable for applications ranging from large-scale population resequencing to reduced-representation genomic analyses. Its potential to revolutionize molecular breeding is particularly notable, as it enables genetic background profiling, high-density linkage mapping, genome-wide association studies (GWAS), cultivar authentication, genomic selection, and transgene monitoring. The extensive versatility of Hyper-seq technology has been demonstrated through its successful application across various plant species. Notable applications include Wang et al.’s (2022) analysis of 150 potato accessions, which identified 10,364 high-confidence SNPs. Subsequent GWAS implementation revealed 58 candidate genes associated with tuber pigmentation. Similarly, Fu et al. (2022) explored 241 *Canna edulis* accessions, detecting a total of 15,659,890 genomic variants (including SNPs and InDels), and identified 550 loci associated with leaf morphology and 240 loci related to color traits. In another related investigation, Ding et al. (2023) examined137 varieties of areca palm (*Areca catechu*), revealing 45,094 SNPs and facilitating association mapping that pinpointed 200 loci related to fruit shape and identified 86 potential regulators of betel nut morphology. Expanding on these applications, Zhou et al. (2024) utilized Hyper-seq technology to analyze 132 *Rubus chingii* germplasm lines, generating 1,303,850 SNPs and 433,159 InDels. Their population genomics study established a core germplasm repository consisting of 38 elite accessions. Collectively, these pivotal studies highlight the remarkable capability of Hyper-seq technology in unraveling genetic architectures and enhancing marker-assisted breeding strategies, positioning it as a transformative tool for accelerating molecular improvement in *Allium* crops and beyond.

In this study, we employed hyper-seq technology to conduct simplified genome sequencing on 100 A*. tuberosum* accessions gathered from various geographic regions throughout China. The sequencing results provided unparalleled resolution for establishing distinct molecular fingerprints across all germplasm analyzed. This study marks the first thorough investigation of genetic diversity in *A. tuberosum*, integrating both SNP and InDel markers within a cohesive analytical framework. Our analysis produced a comprehensive dataset of high-quality SNPs and InDels serving as a vital genetic resource for germplasm identification, resource utilization, and marker-assisted breeding. These results offer fundamental insights to advance molecular genetics research and marker-assisted breeding strategies in *A. tuberosum*.

## Materials and methods

### Plant materials

A total of 100 accessions of *A. tuberosum*, comprising 50 dormant and 50 non-dormant varieties, were chosen for this study. Comprehensive details regarding these accessions can be found in **Supplementary Table 1**. The plants, which were two-year-old at the time of sampling, were cultivated at the Pingdingshan Modern Agricultural Research and Development Base (E113°16’, N33°40’, Henan Province, China). Fresh leaves were harvested from three randomly selected plants for each accession, immediately flash-frozen in liquid nitrogen, and subsequently stored at -80 °C for further analyses.

### DNA extraction and library construction

Genomic DNA was extracted using the Hi-DNAsecure Plant Kit [TIANGEN BIOTECH (BEIJING) Co., LTD] following the manufacturer’s instructions. DNA quality was tested by electrophoresis in 0.8% agarose gel. Its purity and concentration were measured with a Nanodrop^®^ ND-100 UV/V spectrophotometer (Thermo Fisher Scientific, Waltham, MA, USA). To facilitate effective library construction, the extracted DNA was standardized to a concentration between 150ng/μL and 200ng/μL. The library was construction following the Hyper-seq protocol as outlined by Zou and Xia (2022). Once the library passed quality assessment, high-throughput sequencing was conducted using the second-generation platform DNBSEQ-T7 platform (BGI Genomics Co., Ltd). Quality control and filtering of the raw data were performed using Fastp (version: 0.23.4, parameters: Default parameter) (Chen et al., 2018). The clean reads from each sample were then aligned to the reference genome (GCA_030737875.1) using BWA (version: 0.7.17, parameter: mem) (Li and Durbin, 2009).

### Identification of SNPs and InDels

Variants were called using Genome Analysis Toolkit (GATK, version 4.4.0.0) with the HaplotypeCaller, CombineGVCFs, and GenotypeGVCFs tools. Subsequently, hard-filtering was applied using GATK VariantFiltration with plant specific thresholds to obtain high-confidence SNPs and InDels (McKenna et al., 2010). The specific filtering criteria are as follows:

For SNP identification, the criteria are: “QD < 2.0 || QUAL < 30.0 || SOR > 3.0 || FS > 60.0 || MQ < 40.0 || MQRankSum < -12.5 || ReadPosRankSum < -8.0”.

For InDel identification, the criteria are: “QD < 2.0 || QUAL < 30.0 || FS > 200.0 || MQ < 40.0 || ReadPosRankSum < -20.0”.

### Population structure

For the analysis of population structure, the mutation dataset, which had previously been rigorously filtered using the GATK, underwent further quality control with VCFtools (version 0.1.16, parameters: -MAF, -max-missing, min-alleles, max-alleles, remove-indels) (Danecek et al., 2011). This step aimed to eliminate variant sites with minor allele frequencies below 0.05 and genotype deletion rates exceeding 20%. Only SNP mutation sites with two alleles were retained. Following these stringent filtering criteria, the remaining high-quality variants were utilized for subsequent analyses.

The neighbor-joining (NJ) tree was constructed using the NJ methods in PHYLIP (version 3.696, parameter: neighbor). Visualization of the tree file in Newich format was performed with ggtree. For Principal Component Analysis (PCA), GCTA (version: 1.93.2, parameters: -grm, -pca) (Yang et al., 2013)was employed.

For the analysis of population genetic structure, ADMIXTURE (version: 1.3.0, parameters: -cv imputFile K) (Alexander et al., 2009) was utilized, with K values ranging from 2 to 10. The optimal K value was identified based on the cross-validation (CV) error and the maximum likelihood estimate.

### Core collection construction and evaluation

In this research, the Genocore software (https://github.com/lovemun/Genocore) (Jeong et al., 2017) was utilized to identify core collection, with the accuracy of this identification being verified through PCA analysis of both the original and the identified core collection. Ideally, the PCA map for the core collection should mirror the distribution pattern of all materials, thereby validating the screening process. Furthermore, traditional genetic diversity indices including observed heterozygosity (Ho), expected heterozygosity (He), observed alleles number (Na), effective alleles number (Ne), Nei’s diversity index (H), Shannon’s information (I), were computed to evaluate the genetic diversity of both the original and the selected core collection. Random sampling was conducted across core and non-core germplasm groups to ensure representativeness in downstream analyses.

### DNA fingerprinting

Following variant calling, biallelic SNPs with a MAF at least 0.05 and a genotype missing rate of zero were retained. Subsequently, a genetic algorithm-based optimization was employed to determine combinations of multi-locus markers that could differentiate individual samples, thereby creating DNA fingerprints specific to each sample. To visualize the fingerprint profiles, distinct color-coding schemes were utilized to represent different allelic configurations at each locus, allowing for an intuitive interpretation of genetic identities.

## Results

### Hyper-seq and variant identification

The Hyper-seq was performed on 100 accessions of *A. tuberosum* accessions using the Illumina NovaSeq 6000 platform. The raw sequencing data totaled 1020.29 Gb, averaging 71.34 million reads per accession (**Supplementary Table 2**). After filtering out low-quality reads (Q<20), reads containing more than 5 ambiguous bases (“N”), and those shorter than 50bp, a total of 1003.34 Gb of clean data was obtained, corresponding to an average of 71.30 million clean reads per accession. The quality of the clean data was high, with Q30 values exceeding 90.41% and GC content ranging from 40.82% to 44.04%, confirming its appropriateness for subsequent SNP/InDel identification (**Supplementary Table 2**). The clean reads were aligned to the *A. sativum* reference genome (GCA_030737875.1) utilizing BWA-MEM v0.7.17 with default parameters. The mapping efficiency varied between 6.62% to 17.19%, yielding genomic coverage of 0.115–0.411% and an average sequencing depth of 0.007–0.034× (**Supplementary Table 3**).

**Figure 1:**
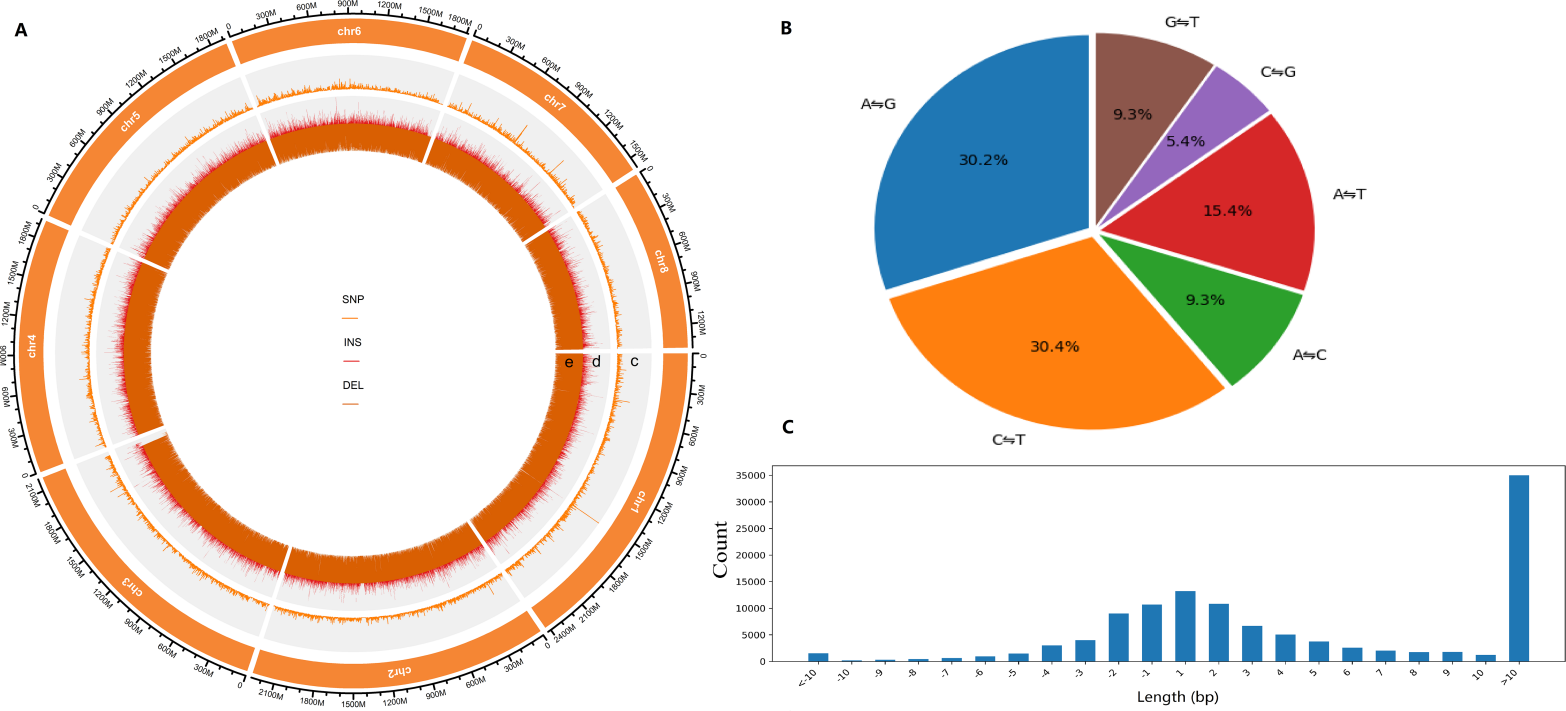
Analysis of SNPs and InDels in Chinese chive genomes. **(A)** Genome-wide variation distribution. c, number of SNPs, d, number of insertions, e, number of deletions. **(B)** Proportion of six variant types of SNPs in the whole population. **(C)** Distribution of InDel lengths.

A total of 299,425 SNPs and 130,325 InDels were detected across eight chromosomes and 950 scaffolds (**Supplementary Table 5**, **Supplementary Figures 1-3**), with both types of variants showing a uniform genomic distribution (**Figure 1A**). Biallelic sites were predominant (95.42%), with allele counts ranging from 2 to 7 (**Supplementary Table S4**). The average density of SNPs and InDels per chromosome was 0.017-0.020 SNPs/kb and 0.008 InDels/kb, respectively (**Supplementary Table 5**). Analysis of InDel length indicated that longer variants (>10 bp) were more common, comprising 30.15% of the total (**Figure 1C**, **Supplementary Table 7**). The number of chromosomal InDels showed a positively correlation with chromosome length; for instance, Chromosome 1, the longest, contained 20,025 InDels, whereas Chromosome 8, the shortest, had only 11,389 InDels (**Supplementary Table 5**).

Transition (Ti: A ⇋G, C ⇋T) polymorphisms were found to be more prevalent than transversion (Tv: A ⇋C, A ⇋T, C ⇋G, G ⇋T) variants, with a genome-wide Ti/Tv ratio of 1.541 (**Supplementary Table 6**). Analysis of single-nucleotide substitution revealed that transitions C ⇋T (30.4%) and A ⇋G (30.2%) were the most common, while the transversion C ⇋G was the least frequent category, comprising 5.4% of all mutation types (**Figure 1B**).

### Population structure

**Figure 2:**
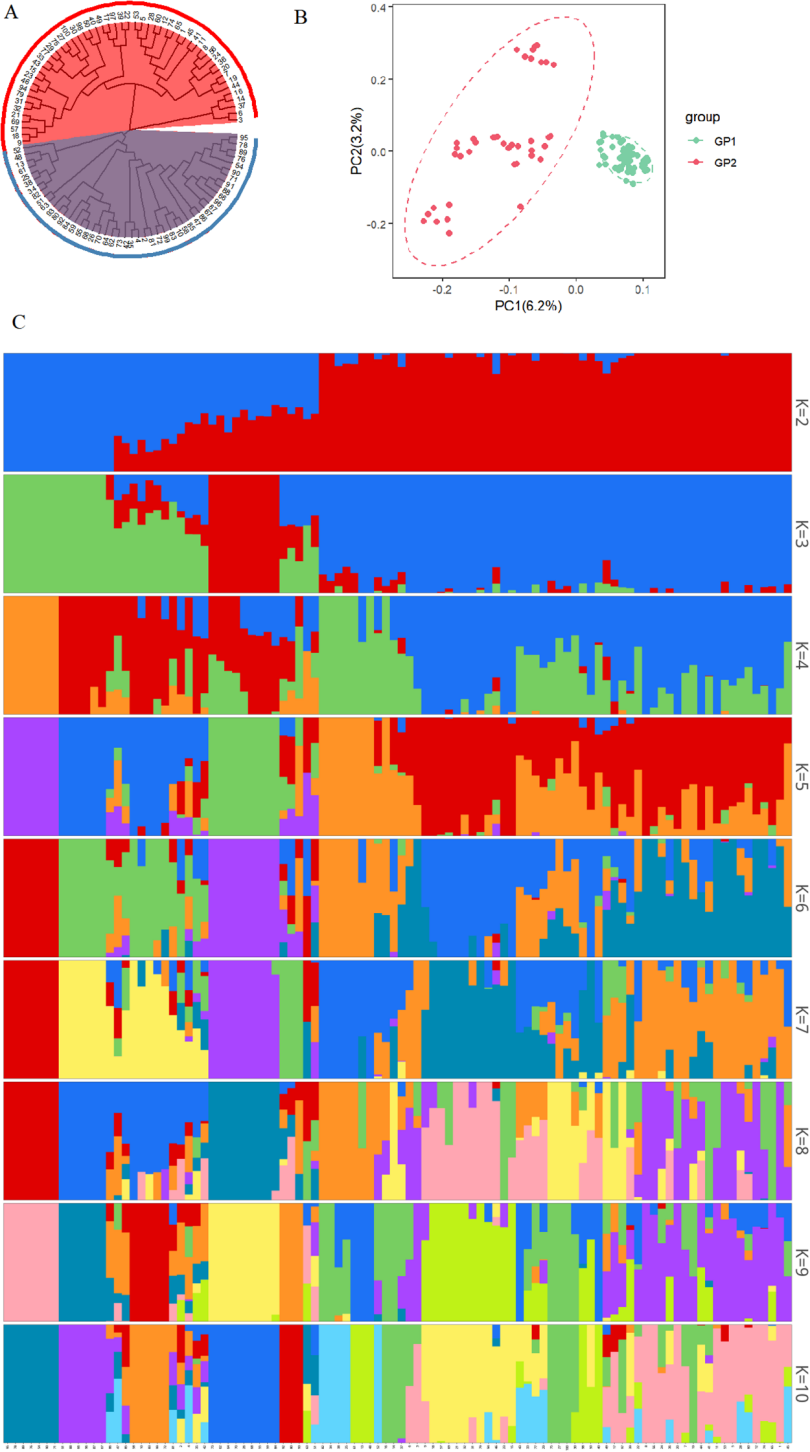
Phylogenetic and population genetic analyses of 100 Chinese chive accessions. **(A)** Neighbor-joining phylogenetic tree based on SNP data. **(B)** Principal component analysis. **(C)** Population structure analysis at K=2-10.

Comprehensive population genomic analyses revealed a distinct bipartite substructure among the 100 Chinese chive accessions. A phylogenetic tree was reconstructed using the NJ method implemented in PHYLIP v3.696 with 1,000 bootstrap replicates. Pairwise squared genetic distances were derived from high-quality SNPs in Variant Call Format (VCF) with a MAF of ≥ 0.05. The resulting NJ tree identified two evolutionarily separate clusters: Group I, comprising 48 accessions (48%), and Group II, consisting of 52 accessions (52%) (**Figure 2A**). In the Principal component analysis (PCA) plot, Principal Component 1 (PC1, accounting for 6.2% of the variance) and Principal Component 2 (PC2, accounting for 3.2% of the variance) effectively separated the accessions into two distinct, non-overlapping clusters (**Figure 2B**, **Supplementary Table 8**). Cross-validation using ADMIXTURE v1.3.0 indicated that K=2 was the most suitable population structure, showing a significantly lower prediction error compared to K values ranging from 3 to 10 (**Figure 2C**, **Supplementary Figure 4**).

### Construction of core collection

**Figure 3:**
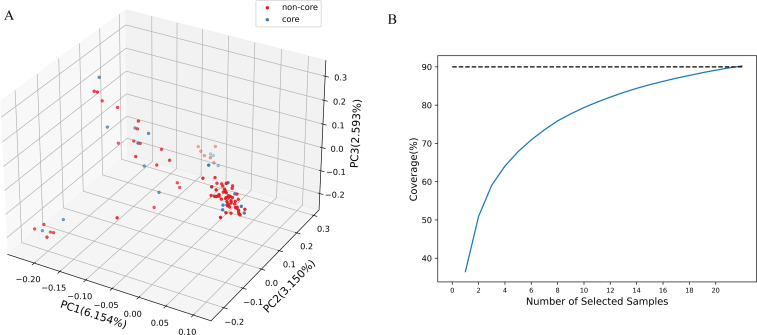
Core collection constructed from 100 Chinese chive accessions. **(A)** Distribution of the core collection within the initial collection. **(B)** Genotype coverage trend map. When the sample size reaches 22, the genotype coverage attains 90.17%.

To effectively manage and utilize genetic resources, a core collection was constructed from 100 Chinese chive accessions using a combination of Hyper-seq technology and Genocore. The core collection comprises 22 accessions, representing 22% of the total, and it retained 90.17% of the original genetic diversity (**Figure 3B**, **Supplementary Table 9**). This result was confirmed by the similar PCA clustering patterns observed for both the core collection and the entire population (**Figure 3A**). Key genetic diversity indices, including Ho, He, Na, Ne, I, and H, were evaluated for both core and non-core collections. The values of these indices were remarkably similar between the two collections (**Table 1**). T-tests showed that the p-values for the comparisons of each index were non-significant (>0.05; ranging from 0.2469 to 0.992), indicating no statistically significant differences (**Supplementary Table 10**). This finding further validates that the core collection has effectively captured the genetic diversity present in the entire population.

**Table 1:** Correlation index of genetic diversity between core and noncore germplasms under different sampling proportions.

Sample population	Sampling proportion	Sample numbers	PLN %	Ho	He	Na	Ne	I	H
Core germplasm	10	2	41.76	0.2170	0.1822	1.4210	1.3305	0.3783	0.3016
Noncore germplasm	10	7	73.99	0.1959	0.2315	1.7399	1.3754	0.5148	0.4857
Core germplasm	25	5	68.37	0.2056	0.2354	1.6837	1.3903	0.5141	0.4596
Noncore germplasm	25	19	86.65	0.2239	0.2543	1.9464	1.4026	0.5793	0.6003
Core germplasm	50	11	84.03	0.2118	0.2585	1.8837	1.4139	0.5813	0.5711
Noncore germplasm	50	39	90.47	0.2266	0.2622	1.9936	1.4125	0.6005	0.6279
Core germplasm	75	16	85.79	0.2173	0.2629	1.9489	1.4155	0.5972	0.6059
Noncore germplasm	75	58	92.19	0.2330	0.2629	2.0000	1.4137	0.6028	0.6315
Core germplasm	100	22	90.17	0.2163	0.2650	1.9833	1.4160	0.6053	0.6241
Noncore germplasm	100	78	94.29	0.2313	0.2634	2.0000	1.4137	0.6047	0.6317

PLN, polymorphic loci numbers.

### Construction of SNP fingerprints

**Figure 4:**
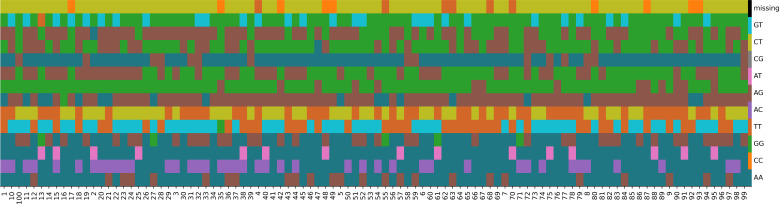
Fingerprints of 100 Chinese chive accessions. Each line represents one SNP locus, and each column represents one accession.

To facilitate the preliminary identification of Chinese chive germplasm resources, a DNA fingerprint was established for these 100 accessions. From the initial pool of 291, 547 SNPs, stringent filtering (MAF≧0.05 and zero missing data) yielded 150 high-quality candidate SNPs. Then a genetic algorithm was applied to optimize marker combinations, minimizing redundancy while maximizing discriminatory power. This iterative process selected 14 diagnostic SNPs capable of distinguishing all accessions (**Supplementary Table 11**), forming a standardized fingerprinting system (**Figure 4**).

## Discussion

Advancements in sequencing technologies and decreasing costs have promoted the widespread adoption of high-density molecular markers such as SNPs, SSRs, and InDels. However, whole-genome resequencing remains financially unfeasible for species with large or complex genomes. Simplified genome sequencing methods, including Reduced representation library sequencing (RRLs), Restriction site Associated DNA (RAD), Genotyping by Sequencing (GBS), Specific Focus Amplified Fragment Sequencing (SLAF), and Double Digest Restriction Associated DNA (ddRAD), offer effective solutions for economical acquisition of numerous molecular markers (Davey et al., 2011; Miller et al., 2007). Notably, Hyper-seq technology introduces a novel PCR-based library preparation strategy, eliminating the need for restriction enzyme digestion and adaptor ligation through streamlined amplification. By adjusting Hyper-seq primers, researchers can efficiently control marker density and perform multiplexed library preparation, enabling ultra-low-cost sequencing in species such as *Canna edulis* (Fu et al., 2022), *Areca catechu* (Ding et al., 2023), and *Rubus chingii* (Zhou et al., 2024).

As an autotetraploid vegetable crop with facultative apomixis (Zhang et al., 2020), *A. tuberosum* faces significant obstacles in molecular marker development due to the lack of a comprehensive reference genome. Previous research focusing on SSR markers was constrained by methodological limitations, primarily relying on transcriptome-derived data (Zhou et al., 2015; Li et al., 2020b). In this groundbreaking study, we utilized Hyper-seq technology to perform streamlined genome sequencing on 100 genetically diverse *A. tuberosum* accessions. Using the *A. sativum* genome (GCA_030737875.1) as a heterologous reference, we detected 291,547 SNPs and 116,223 InDels, with detection rates of 0.018 SNPs/kb and 0.008 InDels/kb, respectively. Compared to the *Rubus chingii* study (Zhou et al., 2024), sequencing results mapped to the garlic genome showed lower alignment rates (6.62%-17.19), coverage (0.115%-0.411%), and average sequencing depth (0.007×-0.034×). The low alignment rate stems from genomic differences between these two congeneric species, while the low coverage is inherent to Hyper-seq as a reduced-representation sequencing technology, which only sequences a small fraction of the genome, compounded by the large genome size of *Allium* species (Hao et al., 2023). Notably, the low average sequencing depth results from its calculation (aligned bases/total genome bases), whereas the actual depth in targeted regions is sufficient for variant detection.

To address the complexity of autotetraploid genomes, we simplified SNP genotyping using a diploid model, retaining only biallelic variants. Autotetraploid genomes exhibit diverse allelic combinations (e.g., CCCC, GCCC, GGCC, GGGC, GGGG), and direct analysis requires specialized algorithms and software, posing significant technical challenges. By simplifying to a diploid system (e.g., GC), the potential true states of heterozygous loci are reduced to two, while homozygous loci remain unaffected, allowing direct application of mature diploid SNP analysis tools, substantially lowering the technical barrier and improving efficiency. This approach also reduces dosage misassignment due to insufficient sequencing depth, preserves presence/absence information of alleles, minimally impacting GWAS results, and remains effective for locating trait-associated loci. In population genetic analysis, while the simplified approach may slightly affect the precision of kinship inference between individuals, it still reveals the overall genetic structure of populations, making it suitable for rapid screening and preliminary studies. This simplification strategy has been applied in autopolyploid crops such as sugarcane (*Saccharum spontaneum*) (Zhang et al., 2018), alfalfa (*Medicago sativa*) (Shen et al., 2020), and potato (*Solanum tuberosum*) (Zhao et al., 2023), demonstrating its generalizability for genomic studies of autopolyploid plants, especially in the absence of high-quality reference genomes or limited sequencing depth.

*A. tuberosum*, a perennial cold-hardy vegetable, displays distinct dormancy patterns categorized into dormant and non-dormant ecotypes based on complete or partial senescence of aboveground foliage during winter. This study selected 100 representative accessions (50 dormant and 50 non-dormant) to investigate population genetic differentiation. Through multivariate analyses including phylogenetic tree, principal component analysis (PCA), and genetic structure assessments, the 100 accessions were grouped into two distinct genetic clusters (Cluster I: 52 accessions, Cluster II: 48 accessions). Intriguingly, this genetic separation did not fully align with prior dormancy classifications: Cluster I predominantly comprised non-dormant accessions (75.0%, 39/52) with a smaller proportion of dormant types (25.0%, 13/52), while Cluster II showed the opposite trend, with 77.1% dormant (37/48) and 22.9% non-dormant (11/48). These results indicate that the dormancy in *A. tuberosum* is a quantitative trait influenced by genetic background, involving multiple genes or gene-environment interactions, with a more complex genetic mechanism than simple ecotypic classification. Plant dormancy is a complex trait influenced by photoperiod and temperature, involving intricate physiological and biochemical changes regulated by biological clocks, plant hormones, and epigenetic mechanisms (Zhang et al., 2019), serving as a survival strategy to withstand harsh environmental conditions such as extreme temperatures, diurnal variations, and nutrient scarcity (Horvath et al., 2003).

In agricultural research, core collections are vital for preserving genetic diversity in compact germplasm subsets, enhancing resource management efficiency and facilitating global germplasm exchange while safeguarding essential genetic information. Using Genocore software, we established a core collection of 22 representative germplasms (22% of the total) from 100 A*. tuberosum* accessions, retaining 90.17% of the original polymorphism sites. Genetic diversity indices of the core collection showed no significant difference from the non-core collection, confirming effective preservation of genetic diversity (**Supplementary Table 10**). These findings highlight the substantial genetic variation retained in the selected subset, underpinning its potential for molecular-assisted breeding and genomic selection in *A. tuberosum* improvement. Previous studies show core collections typically encompass 5-40% of the original accessions, with optimal sampling intensity for most crop ranging from 5% to 15%, such as sesame (*Sesamum indicum*, 9.98%, 501/5020; Liu et al., 2017), Chinese raspberry (*Rubus chingii*, 28.8%, 38/132; Zhou et al., 2024), radish (*Raphanus sativus*, 19.8%, 43/217; Li X. et al., 2023), watermelon (*Citrullus lanatus*, 10.86%, 130/1197; Zhang et al., 2016), water lily (*Nymphaea* spp., 15%, 36/240; Su et al, 2023), and rice (*Oryza sativa*, 17.3%, 520/3004; Kumar et al., 2020). A widely accepted guideline suggests core subsets should ideally include a minimum 20 accessions to ensure sufficient genetic representation (Franco-Duran et al., 2019). The establishment of a core collection for *A. tuberosum* provides a crucial basis for improving germplasm resource management and utilization. Future research will prioritize two key areas: enhancing systematic evaluations and deep genetic characterization of the established core collection to identifying functional genes associated with important agronomic traits, and continuously integrating newly acquired *A. tuberosum* germplasm resources to refine and diversify the core collection, fostering a solid foundation for developing innovative varieties and advancing breeding programs.

DNA fingerprinting, a DNA-level technique for individual identification using molecular markers, is crucial for genetic diversity analysis, variety identification, authenticity verification, genetic relationship determination, agronomic trait association, and variety right protection. Common molecular markers include SNPs (Single Nucleotide Polymorphisms), SSRs (Simple Sequence Repeats), InDels (Insertion/Deletion variations), ISSR (Inter-Simple Sequence Repeat Analysis), and AFLP (Amplified Fragment Length Polymorphism) (Xing et al., 2024b; Zhang et al., 2024), among which only SNPs and SSRs are endorsed by the International Union for the Protection of New Varieties of Plants (UPOV) in its Biochemical and Molecular Techniques (BMT) guidelines (Jamali et al., 2019). Compared to conventional markers, SNPs offer advantages of high genomic abundance, extensive genome-wide distribution, inherent stability and heritability, and simplified detection, enabling efficient high-throughput genotyping. An increasing number of plant species, such as cauliflower (*Brassica oleracea*) (Yang et al., 2022), radish (*Raphanus sativus*) (Xing et al., 2024a), tobacco (*Nicotiana tabacum*) (Wang et al., 2021), sugarcane (Zhang et al., 2022), sweet potato (*Ipomoea batatas*) (Luo et al., 2023), and honeysuckle (*Lonicera japonica*) (Li J. et al., 2023), are using SNP markers to construct detailed fingerprint profiles, enhancing resource management and variety protection. In this study, we constructed a DNA fingerprint profile for 100 A*. tuberosum* accessions using 14 SNP loci, aimed at identifying homonymous (same name, different genotypes) and synonymous (different names, same genotype) accessions. Accordingly, further validation of these SNP loci was not conducted in this study. In follow-up research, we will validate the identified SNP/InDel loci and apply them to variety purity evaluation, new variety right protection, molecular marker-assisted selection breeding, and functional gene mapping (e.g., genes related to dormancy traits).

## Conclusion

This research has shown that Hyper-seq technology serves as an exceptionally effective approach for the development of SNP and InDel markers in *A. tuberosum* accessions. These markers can be employed to explore population structure and genetic diversity, create a core collection, and establish DNA fingerprinting profiles. By utilizing Hyper-seq technology on 100 A*. tuberosum* accessions sourced from various geographical regions across China, we detected 291,547 SNPs and 116,223 InDels polymorphic loci. With these loci, we divided the accessions into two distinct subgroups. Interestingly, this genetic classification did not completely correspond with the classifications based on dormant phenotypic characteristics. Additionally, we formed a core germplasm collection consisting of 22 accessions, achieving a genotype coverage of 90.17%, and established a DNA fingerprinting system for all 100 accessions using 14 high-quality SNP markers. These results provide a solid foundation for the genotyping, classification, and accurate identification of *A. tuberosum* germplasm resources in breeding initiatives.

## Data Availability

The original contributions presented in the study are publicly available. Raw sequencing data have been deposited in the National Genomics Data Center (NGDC) (https://ngdc.cncb.ac.cn/) under the accession number PRJCA038045.
