# Supplementary figures and images for: Construction of a core collection and SNP fingerprinting database for Chinese chive (Allium tuberosum) through Hyper-seq based population genetic analysis
Source: Front Plant Sci. 2025 Jul 16;16:1603210. doi: 10.3389/fpls.2025.1603210 (PMC12307429; doi:10.3389/fpls.2025.1603210)

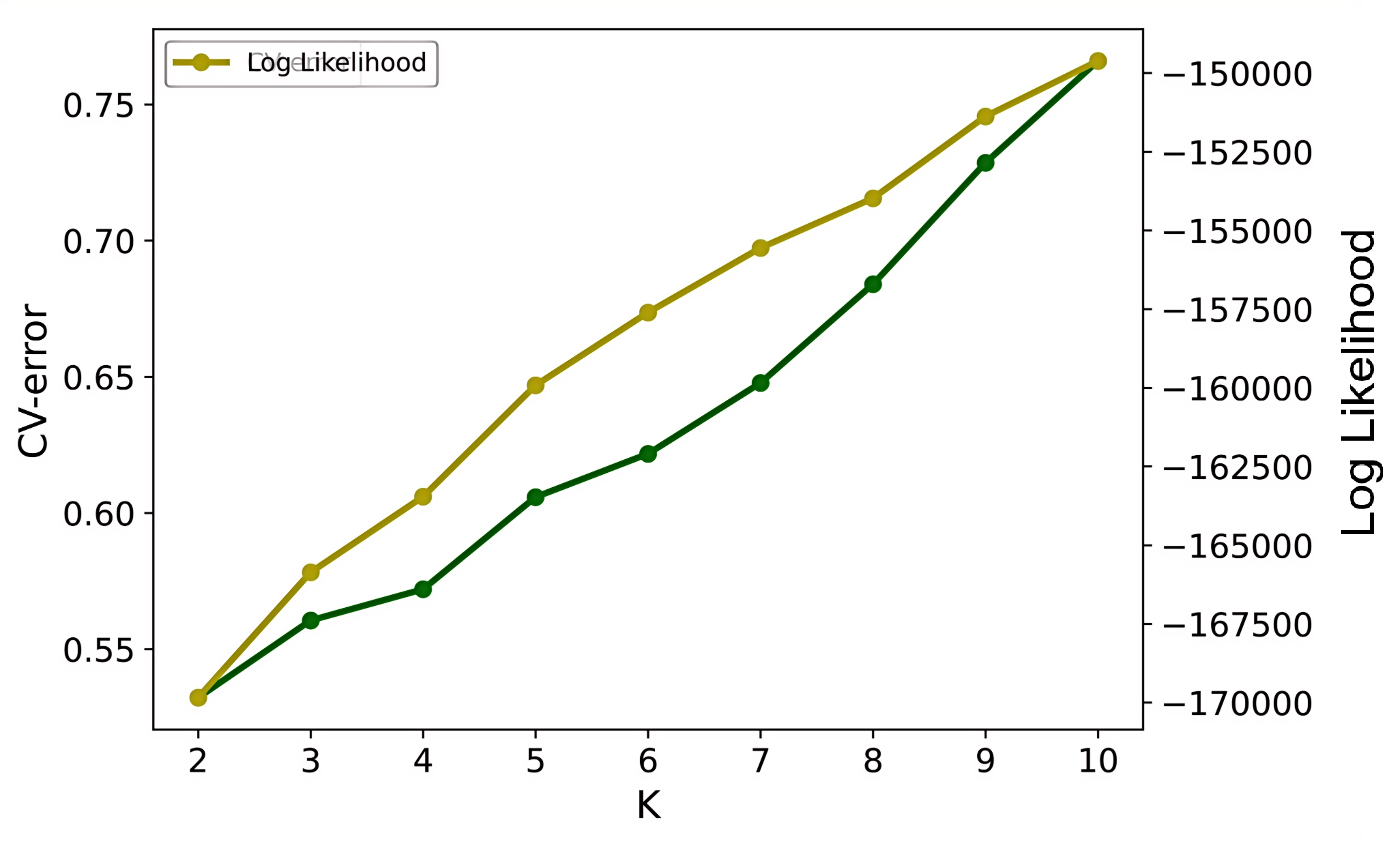

Supplement: Supplementary file 5 [file Image4.tif]
